# Genetic mapping of a single nuclear locus determines the white flesh color in watermelon (*Citrullus lanatus* L.)

**DOI:** 10.3389/fpls.2023.1090009

**Published:** 2023-02-07

**Authors:** Licong Yi, Wei Zhou, Yi Zhang, Zibiao Chen, Na Wu, Yunqiang Wang, Zhaoyi Dai

**Affiliations:** ^1^ Hubei Key Laboratory of Vegetable Germplasm Enhancement and Genetic Improvement, Industrial Crops Institute, Hubei Academy of Agricultural Science, Wuhan, China; ^2^ Key Laboratory of Ecological Cultivation on Alpine Vegetables (Co-construction by Ministry and Province), Ministry of Agriculture and Rural Affairs, Wuhan, China; ^3^ Hubei Key Laboratory of Optical Information and Pattern Recognition, Wuhan Institute of Technology, Wuhan, China; ^4^ College of Horticulture and Gardening, Yangtze University, Jingzhou, China

**Keywords:** watermelon, genetic mapping, flesh color, carotenoids, chromoplast

## Abstract

**Introduction:**

Flesh color is an important trait in watermelon (Citrullus lanatus L.). Several flesh color genes have been identified in watermelon; however, the inheritance of and the molecular basis underlying the white flesh trait remain largely unknown.

**Methods:**

In this study, segregation populations were constructed by crossing the canary yellow flesh line HSH-F with the white flesh line Sanbai to fine-map the white flesh gene in watermelon.

**Results:**

Genetic analysis indicated that the white flesh trait is controlled by a single recessive locus, termed Clwf2. Map-based cloning delimited the Clwf2 locus to a 132.3-kb region on chromosome 6. The candidate region contains 13 putative genes, and four of them—Cla97C06G121860, Cla97C06G121880, Cla97C06G121890, and Cla97C06G121900—were significantly downregulated in the white flesh compared to the canary yellow flesh watermelon fruits. The Cla97C06G121890 gene, which encodes a tetratricopeptide repeat protein, showed almost no expression in the white flesh fruit before maturity, whereas it had a very high expression in the canary yellow flesh fruit at 18 days after pollination. Transmission electron microscopy revealed rounded and regularly shaped chromoplasts in both the canary yellow and white flesh fruits. Further quantitative real-time PCR analysis showed that the expression levels of several key plastid division genes and almost the entire carotenoid biosynthesis pathway genes were downregulated in the white flesh compared to the canary yellow flesh fruits.

**Discussion:**

This study suggests that the proliferation inhibition of chromoplasts and downregulation of the CBP genes block the accumulation of carotenoids in watermelon and lead to white flesh. These findings advance and extend the understanding of the molecular mechanisms underlying white flesh trait formation and carotenoid biosynthesis in watermelon.

## Introduction

Watermelon (*Citrullus lanatus* L., 2*n* = 2*x* = 22) is one of the most popular summer fruits grown all over the world, and it provides rich nutrients in the human diet, including carotenoids, citrulline, and vitamins. Carotenoids are important secondary metabolites that yield the red, orange, and yellow colors in many fruits, flowers, and vegetables (Nisar et al., 2015). In watermelon, the red, orange, and yellow flesh colors are abundant in lycopene, β-carotene, and xanthophyll, respectively (Zhao et al., 2013; Lv et al., 2015). Investigating inheritance of the flesh color and the molecular mechanisms underlying carotenoid accumulation is of significant importance for watermelon breeding.

There are a variety of flesh colors in watermelon, including white, pale yellow, salmon yellow, canary yellow, orange, pink, red, and scarlet red. Researchers have been investigating the inheritance of flesh color in watermelon since 1937 (Porter, 1937), and several flesh color loci have been identified (Poole, 1944; Henderson, 1989; Henderson et al., 1998; Gusmini and Wehner, 2006; Bang et al., 2007; Bang et al., 2010). It has been reported that the *C* and *I* loci determine the canary yellow and red flesh colors in watermelon (Poole, 1944). A study by Henderson et al. (1998) showed that the canary yellow flesh (*C*) is dominant over the red flesh (*c*), but can be inhibited by the homozygous recessive *i* locus, which will result in a red flesh regardless of the *C* alleles present (Henderson et al., 1998). Moreover, Shimotsuma (1963) suggested that the yellow flesh (*B*) and white flesh (*Wf*) loci, with epistatic interaction, control the yellow, red, and white flesh colors in watermelon. Yellow flesh (*B*) is dominant over red flesh (*b*), and *Wf* is epistatic to the yellow and red flesh colors. However, whether *C* and *B* belong to the same locus is still unknown. The red, orange, and salmon yellow flesh colors are controlled by the *Y* locus, which has three alleles, in which *Y* (red flesh) is dominant over both *y°* (orange flesh) and *y* (salmon yellow flesh), and *y°* (orange flesh) is dominant over *y* (salmon yellow flesh) (Henderson, 1989).

With the development of high-throughput sequencing technology and the publication of high-quality watermelon reference genome sequence (Guo et al., 2012; Guo et al., 2019), great breakthroughs have been made in the mapping and cloning of the flesh color genes in watermelon. Branham et al. (2017) mapped a major codominant quantitative trait locus (QTL) (i.e., *QFC.1*) that is associated with the accumulation of β-carotene on watermelon chromosome 1 based on linkage analysis. Zhang et al. (2017) revealed that the expression of the phosphorus transporter *ClPHT4;2* is essential for pigment accumulation in watermelon. Guo et al. (2019) identified five flesh color loci in watermelon using genome-wide association study (GWAS), in which the *FC4.1* locus on chromosome 4 contains a lycopene β-cyclase (*LCYB*) gene. Mutation in the *LCYB* gene has been reported to lead to flesh color changes from lemon yellow to red (Bang et al., 2007). Li et al. (2020) identified a locus (i.e., *Y^scr^*) on chromosome 6 that determines the scarlet red flesh color trait in watermelon. Diao et al. (2021) located a locus for the canary yellow flesh color on chromosome 6. In addition, Wang et al. (2021) mapped a locus associated with the pale yellow and white flesh color variations on chromosome 1. The expression level of *ClPSY1* was positively correlated with lycopene accumulation in watermelon (Guo et al., 2019), and a nucleotide variation in the *ClPSY1* gene resulted in a golden flesh color (Liu et al., 2022). Furthermore, comparative transcriptome studies showed that low expression of the lycopene cyclase genes (*LCYB/LCYE*) during fruit development is a key factor leading to lycopene accumulation in watermelon (Grassi et al., 2013; Lv et al., 2015).

The carotenoid biosynthesis pathway (CBP) in plants has been extensively studied, and the major genes and enzymes in this pathway have been well characterized (Cazzonelli and Pogson, 2010; Nisar et al., 2015; Yuan et al., 2015; Sun et al., 2018). The C_20_ glyceraldehyde 3-phosphate (GGPP) molecule, which is produced from isopentenyl diphosphate (IPP) and dimethylallyl diphosphate (DMAPP) of the methylerythritol 4-phosphate (MEP) pathway, is the immediate precursor of carotenoids (Fraser and Bramley, 2004). *PSY* catalyzes the condensation of two GGPP molecules to form C_40_ carotenoid phytoene. Phytoene is desaturated and isomerized to produce lycopene under catalysis of phytoene desaturase (PDS), ζ-carotene dehydrogenase (ZDS), ζ-carotene isomerase (Z-ISO), and ζ-carotene desaturase (CRTISO) (Nisar et al., 2015). Lycopene is cyclized by *LCYB* and *LCYE* to produce β-carotene and α-carotene, respectively. These carotenes are further converted to xanthophylls through a series of hydroxylation reactions. In addition to the CBP genes, several transcriptional regulators, such as MYB, WRKY, MADS, bHLH, B-box, ERF, and NAC, that control the accumulation of carotenoids by directly interacting with the CBP genes to repress or activate their expression have been identified (Welsch et al., 2007; Ma et al., 2014; Larkin et al., 2016; Sagawa et al., 2016; Fu et al., 2017; Lu et al., 2018; Ampomah-Dwamena et al., 2019; Meng et al., 2019; Xiong et al., 2019; Zhou et al., 2019; Duan et al., 2022). For example, WHITE PETAL1 (WP1), a R2R3-MYB protein, plays a crucial role in regulating floral carotenoid pigmentation in *Medicago* by directly regulating the expression of *MtLYCe* and *MtLYCb* (Meng et al., 2019). In kiwifruit, *TFMYB7* controls pigment accumulation during fruit ripening by activating the promoter of the *AdLCYb* gene (Ampomah-Dwamena et al., 2019). In tomato, *SlNAC1* interacts with the *SlPSY1* gene to alter the carotenoid pathway flux (Ma et al., 2014).

Colored flesh watermelon cultivars evolved from white flesh wild watermelon, and human selection of CBP genes, particularly the *PSY* and *LCYB* genes, have played a key role in flesh coloration in watermelon (Guo et al., 2019). In the present study, we report the mapping and candidate gene prediction of the *Clwf2* locus that determines the white flesh trait in watermelon. Genes related to plastid proliferation and carotenoid biosynthesis were significantly downregulated in white flesh watermelon fruits. The findings of this study provide new insights into flesh color formation and promote understanding of carotenoid accumulation in watermelon.

## Materials and methods

### Plant materials and genetic populations

The watermelon germplasms HSH-F, Sanbai, and CG149 are preserved by the Industrial Crops Institute of Hubei Academy of Agricultural Sciences, Wuhan. The flesh colors of these germplasms are canary yellow, white, and orange, respectively.

For genetic inheritance analysis and genetic mapping of the white flesh trait, the canary yellow flesh inbred line HSH-F (P_1_) was crossed with the white flesh line Sanbai (P_2_) to produce F_1_ hybrids. Subsequently, the F_1_ plants were backcrossed with HSH-F and Sanbai to produce the BC_1_P_1_ and BC_1_P_2_ populations and were self-pollinated to generate the F_2_ segregation population. F_2_ individuals were further self-pollinated and individually harvested to generate F_3_ families. The F_2_ segregation population was used for genetic mapping of the white flesh gene. To examine whether the white and canary yellow flesh traits are controlled by the same locus, the orange flesh line CG149 (P_3_) was crossed with HSH-F and Sanbai to produce the F_1_(P_3_×P_1_) and F_1_(P_3_×P_2_) hybrids, respectively. The plant materials were creeping-cultured in a greenhouse under natural light conditions at the vegetable test base of Hubei Academy of Agricultural Sciences, Wuhan (30°36′ N, 104°18′ E). The greenhouse was maintained at daily temperatures between 20°C and 40°C and relative humidity between 50% and 85%. Female flowers were pollinated from 7:00 to 9:00 a.m. The flesh colors of the watermelon fruits were visually recorded at 34 days after pollination (DAP).

### Genome resequencing and variant calling

Genomic DNA was extracted from the leaves of HSH-F and Sanbai using the cetyltrimethyl-ammonium bromide (CTAB) method (Porebski et al., 1997). The integrity of genomic DNA was examined using 1% agarose gel, purity was assessed with a NanoDrop2000 spectrophotometer (Thermo Fisher Scientific, Waltham, MA, USA), and the concentrations were determined using Qubit 3.0 (Thermo Fisher Scientific). A DNA library was constructed using the NEB_Next Ultra II DNA Library Prep Kit for Illumina (New England Biolabs, Hitchin, UK) according to the manufacturer’s instructions and then sequenced using the HiSeq 4000 (Illumina, San Diego, CA, USA) platform with a paired-end run (2 × 150 bp). After removing the adaptors and low-quality reads, the clean reads were mapped to the watermelon reference genome 97103 (v2) (http://cucurbitgenomics.org/organism/21) using the Burrows–Wheeler Aligner (BWA, v0.7.15-r1140) software. The Genome Analysis ToolKit (GATK, v3.7) was used for single nucleotide polymorphism (SNP) and insertion–deletion (InDel) calling.

### Genetic analysis of the flesh color trait

For genetic inheritance analysis, the F_2_ (*n* = 137), BC_1_P_1_ (*n* = 39), and BC_1_P_2_ (*n* = 41) segregation populations derived from the cross between HSH-F and Sanbai were grown in the spring of 2019, and the flesh colors were recorded individually at 34 DAP. To validate the genetic inheritance of flesh color, 51 F_3_ families were grown in the fall of 2019 for phenotyping and segregation analysis. For each F_3_ family, the flesh colors of 15 individuals were recorded. The deviation from the expected segregation ratio of each population was analyzed using the chi-square test.

### Marker development and genetic mapping

For preliminary mapping of the white flesh locus, an SNP panel containing 390 SNPs distributed evenly across 11 watermelon chromosomes was developed based on published watermelon resequencing data (ftp://cucurbitgenomics.org/pub/cucurbit/reseq/watermelon/v2/). The primer sequences and genomic positions of the 390 SNPs are listed in **Supplementary Table S1**. The F_2_ population with 137 individuals and the 51 F_3_ families derived from the cross between HSH-F and Sanbai were firstly genotyped using the SNP panel. Based on the phenotypes of the F_2_ and F_3_ populations, QTL analysis was performed by composite interval mapping (CIM) using WinQTLCart2.5 software (Li et al., 2007). The logarithm of the odds (LOD) threshold was set at 2.5. To narrow the QTL region (Chr06:15.92–24.50 Mb), six InDel markers (**Supplementary Table S1**) with an average density of approximately 1.2 Mb were designed and used to genotype the 137 F_2_ individuals.

According to the preliminary mapping results, two flanking InDel markers were used to screen recombinant plants from the 1,054 F_2_ individuals at the seedling stage in the spring of 2020. These recombinants were further transplanted in the greenhouse for phenotyping. Seven SNP markers (**Supplementary Table S1**) with an average density of about 100 kb were designed and used to genotype the recombinant plants in order to fine-map the white flesh locus.

### Functional annotation of candidate genes

Functional annotation of the genes in the fine-mapped region was based on watermelon reference genome 97103 (v2) (ftp://cucurbitgenomics.org/pub/cucurbit/reseq/watermelon/v2/).

### Quantitative real-time PCR analysis

Flesh samples from the HSH-F and Sanbai lines were collected from the center of three parental fruits at 10, 18, 26, and 34 DAP. The flesh tissues were immediately frozen in liquid nitrogen and stored at −80°C for RNA extraction. Total RNA was extracted using an RNAprep Pure Plant Kit (BioTeKe, Beijing, China) according to the manufacturer’s instructions. The concentrations of RNA were determined using NanoDrop spectrometry. Reverse transcription was performed using a RevertAid First Strand cDNA Synthesis Kit (Thermo Fisher Scientific). The expression levels of the candidate genes were determined by quantitative real-time PCR (qRT-PCR) assays using the MonAmp Fast SYBR Green qPCR Mixture (Monad, Wuhan, China) with the QuantStudio 5 system (ABI, Foster City, CA, USA). The *ClACT* (*Cla97C02G026960*) gene was used as an internal reference. The relative expression levels were calculated using the 2^−(ΔΔ^ *^C^* ^t)^ method (Livak and Schmittgen, 2001). Each test had three biological and technical replicates. The genes and primers used for qRT-PCR analysis are listed in **Supplementary Table S1**. Student’s *t*-test was used to assess statistical differences, and a *p*-value of 0.05 was used to determine the significance of the differences between means.

### Detection of carotenoids

Flesh samples from the HSH-F, Sanbai, and CG149 lines were collected from the center of three parental fruits at 34 DAP. The flesh tissues were cut into small pieces, immediately frozen in liquid nitrogen, and then stored at −80°C. Subsequently, the samples were freeze-dried, ground into powder (30 Hz, 1.5 min), and stored at −80°C until use. About 50 mg powder was weighed and extracted with 0.5 ml mixed solution of *n*-hexane/acetone/ethanol (1:1:1, *v*/*v*/*v*) and the extract vortexed for 20 min at room temperature. Supernatants were then collected after centrifugation at 12,000 rpm for 5 min at 4°C. The residue was re-extracted by repeating the above steps under the same conditions, evaporated to dryness, and reconstituted in a mixed solution of methyl alcohol/methyl *tert*-butyl ether (1:1, *v*/*v*). The solution was filtered using a 0.22-μm membrane filter for further liquid chromatography–tandem mass spectrometry (LC-MS/MS) analysis. The carotenoid content was determined using MetWare (http://www.metware.cn/) based on the AB SciexQTRAP 6500 LC-MS/MS platform. Three biological repeats were performed for each line.

### Transmission electron microscopy of chromoplasts

Transmission electron microscopy (TEM) was used to observe the morphology and ultrastructure of the chromoplasts in the fruits of HSH-F and Sanbai at 34 DAP. Flesh samples from the central areas were cut into 2 mm × 2 mm pieces and immediately fixed with 2.5% glutaraldehyde. After embedding in Spurr’s resin, ultrathin sections of the samples were obtained with a Leica EM UC6 ultramicrotome (Leica Microsystems, Wetzlar, Germany). The sections were examined and photographed with an H-7650 transmission electron microscope (Hitachi, Tokyo, Japan) on the electron microscopy platform of Huazhong Agricultural University.

## Results

### White flesh accumulates trace amounts of carotenoids

**Figure 1:**
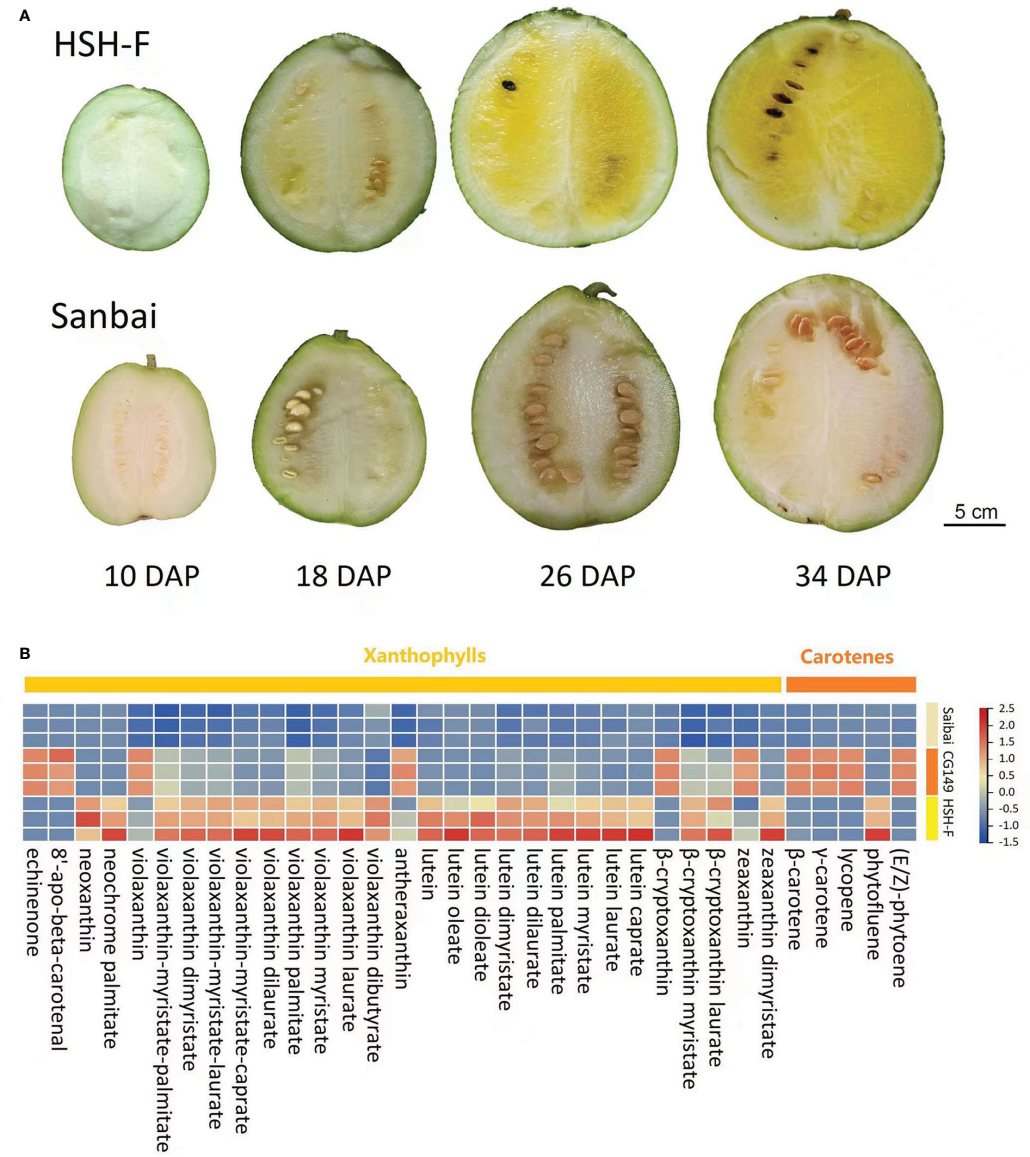
Characterization of the flesh color formation and carotenoid content of parental lines. **(A)** Fruits at 10, 18, 26, and 34 days after pollination (DAP) of HSH-F and Sanbai. **(B)** Heatmap of the carotenoid content of fruits from the parental lines at 34 DAP.

The flesh color of the two parents, HSH-F and Sanbai, was observed at 10, 18, 26, and 34 DAP (**Figure 1A**). Pigment accumulation could be visually observed in the canary yellow flesh parent at 18 DAP, and a distinguishable flesh color was observed in the canary yellow and white flesh parents at 26 DAP. The LC-MS/MS-based determination of the carotenoid content in the fruits of the two parents at 34 DAP showed that the canary yellow flesh contains abundant xanthophyll, whereas the white flesh contains trace amounts of carotenoids (**Figure 1B** and **Supplementary Table S2**). In the canary yellow flesh, 30 types of carotenoids were identified, and most of these were xanthophyll. The five most abundant carotenoids in the canary yellow flesh were violaxanthin-myristate-laurate (7.68 ± 1.80 µg/g), violaxanthin palmitate (6.41 ± 2.50 µg/g), lutein (6.35 ± 1.49 µg/g), violaxanthin myristate (4.77 ± 1.32 µg/g), and violaxanthin-myristate-caprate (2.96 ± 1.24 µg/g) (**Supplementary Table S2**). In white flesh, 19 types of carotenoids were identified, but the carotenoid content was extremely low (**Supplementary Table S2**).

### The white flesh trait is controlled by a single nuclear locus and is epistatic to the canary yellow flesh trait

**Figure 2:**
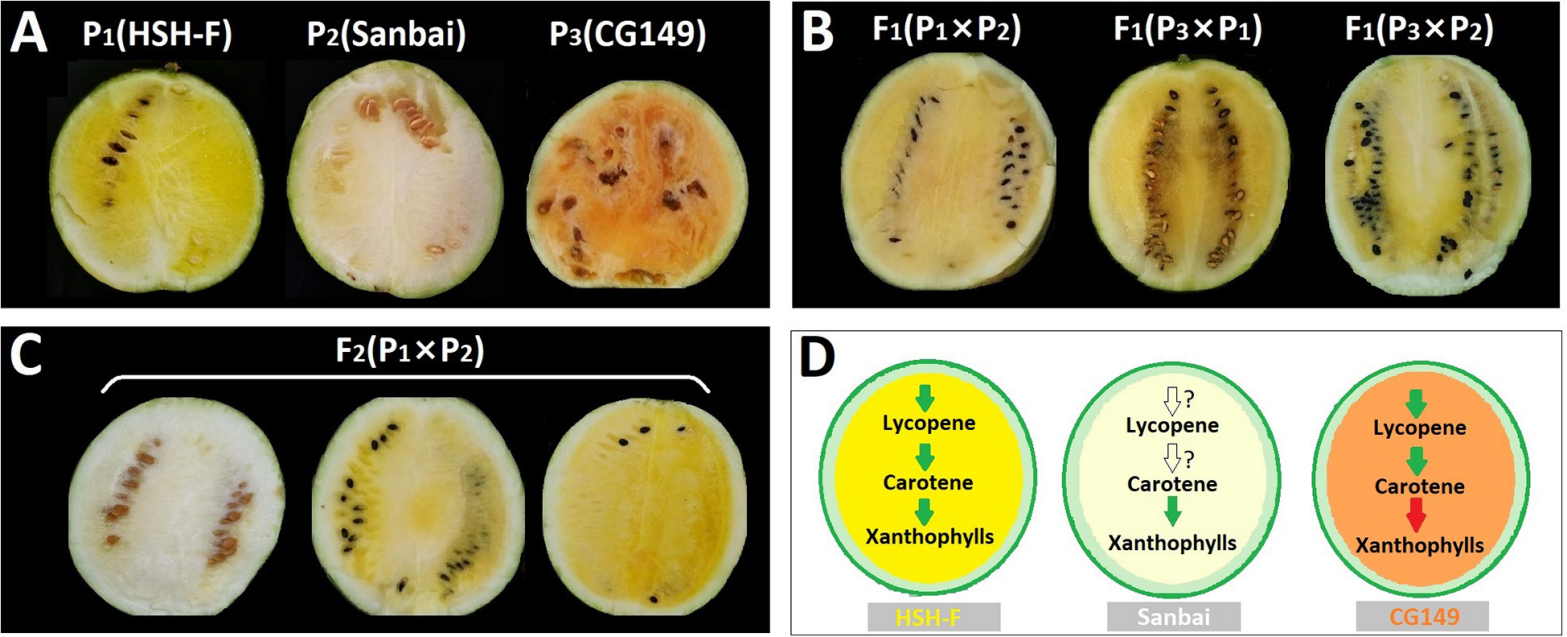
**(A–C)** Phenotype of the flesh colors of the parental lines **(A)**, F_1_ hybrids **(B)**, and the F_2_ segregation population **(C)** at 34 days after pollination (DAP). **(D)** Schematic of the key steps in the carotenoid biosynthesis pathway in the three parents. *Green arrows* indicate normal function, *red arrows* indicate function deficit, and *white arrows* indicate unknown function.

Previous studies have implied an interaction effect between the white flesh and yellow flesh loci (Henderson, 1989; Henderson et al., 1998). In this study, three parents with canary yellow (HSH-F, P_1_), white (Sanbai, P_2_), and orange (CG149, P_3_) flesh colors (**Figure 2A**) were used for genetic analysis of the flesh color in watermelon. Determination of the carotenoid content revealed that carotenes were highly abundant in the orange flesh parent CG149, which lacked xanthophyll (**Figure 1B** and **Supplementary Table S2**). The three most abundant carotenes in CG149 were (E/Z)-phytoene (42.94 ± 1.46 µg/g), lycopene (41.65 ± 3.74 µg/g), and β-carotene (37.00 ± 1.40 µg/g) (**Supplementary Table S2**).

The canary yellow flesh trait was observed in the fruits of the F_1_ hybrids produced from crossing the canary yellow with the white flesh parents (HSH-F × Sanbai) (**Figure 2B**). The F_2_ segregation population derived from the cross between HSH-F and Sanbai showed pure yellow, asymmetrical yellow, and white flesh (**Figure 2C**). Both pure and asymmetrical canary yellow individuals were classified into the yellow flesh group. In the F_2_ population of 137 individuals, 106 plants showed yellow flesh and 31 showed white flesh, confirming a 3:1 Mendelian ratio (*χ* ^2^ = 0.199, *p* = 0.656) (**Table 1**). In the F_3_ population of 51 families, 40 families showed canary yellow or segregated flesh color and 11 families showed white flesh color, which also fitted the expected Mendelian ratio of 3:1 (*χ* ^2^ = 0.154, *p* = 0.699). All 39 individuals in the BC_1_P_1_ population had canary yellow flesh. In the BC_1_P_2_ population of 41 individuals, 16 showed canary yellow flesh and 25 showed white flesh, fitting the Mendelian ratio of 1:1 *(χ* ^2^ = 0.988, *p* = 0.32). Thus, the flesh color variation in this biparental population is controlled by a single nuclear locus.

**Table 1:** Segregation ratio of the flesh color in the F_2_, F_3_, and BC_1_ populations derived from crossing the white flesh with the canary yellow flesh inbred line (HSH-F × Sanbai).

Generation	Total	Yellow	White	Expected (observed) segregation ratio	*χ^2^* value	*p*-value
P_1_ (HSH-F)	10	10	0			
P_2_ (Sanbai)	8	0	8			
F_1_ (P_1_ × P_2_)	6	6	0			
F_2_	137	106	31	3:1 (0.77:0.23)	0.199	0.656
F_2:3_	51	40^a^	11^b^	3:1 (0.78:0.22)	0.154	0.699
BC_1_P_1_	39	39	0	–		–
BC_1_P_2_	41	16	25	1:1 (0.39:0.61)	0.988	0.32

a Fifteen individuals for each family, including all yellow flesh color or segregated flesh families

b All white flesh families.

*χ^2^*, Chi-square test.

To confirm whether the white flesh results from the malfunction of the canary yellow flesh locus, the canary yellow and white flesh parents were crossed with the orange flesh parent (CG149). Both F_1_ hybrids (CG149 × HSH-F and CG149 × Sanbai) showed a canary yellow flesh color (**Figure 2B**). Thus, the white flesh line Sanbai complemented the malfunction in xanthophyll biosynthesis in the orange flesh line CG149. Hence, the genes involved in xanthophyll biosynthesis in the white flesh parent might function normally (**Figure 2D**). These results indicated that the white flesh trait in watermelon is controlled by a single recessive locus, i.e., *Clwf2*, and is epistatic to the canary yellow flesh.

### *Clwf2* is located on chromosome 6

**Figure 3:**
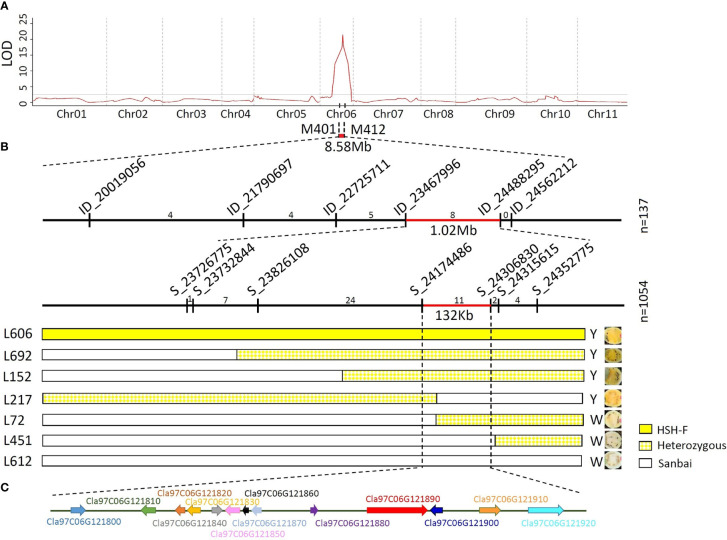
Genetic mapping of the *Clwf2* locus. **(A)** Preliminary mapping of the *Clwf2* locus. **(B)** Fine mapping of the *Clwf2* locus. **(C)** Genes in the candidate region for *Clwf2*. *Numbers above the chromosome* represent recombinants between adjacent markers; *n* is the number of plants used for mapping.

For preliminary mapping of the white flesh locus, the SNP panel containing 390 SNP markers (**Supplementary Table S1**) was used to identify the genotype of the 137 F_2_ individuals and the 51 F_3_ families. Based on the phenotypic data of the F_2_ population and the F_3_ families, a locus associated with variations in flesh color was mapped to the watermelon chromosome 6 in an 8.58-Mb region (**Figure 3A**) between the markers M401 (15.92 Mb) and M412 (24.50 Mb). The highest LOD scores for the 2019 F_2_ and 2019 F_3_ populations were 21.43 and 13.22, which explained 65.543% and 63.55% of the phenotypic variation, respectively (**Supplementary Table S3**).

To fine-map the *Clwf2* locus, the genomes of the parental lines HSH-F and Sanbai were resequenced using the Illumina HiSeq platform for marker development. A total of 96,524 variants were identified between the two parents, including 86,230 SNPs and 10,294 InDels (**Supplementary Table S4**). Firstly, six InDel markers were designed and used to identify the genotype of the 137 F_2_ individuals used for preliminary mapping. This narrowed the *Clwf2* locus in the region of 1.02 Mb between markers ID_23467996 (23.47 Mb) and ID_24488295 (23.49 Mb) (**Figure 3B**). The markers ID_23467996 and ID_24488295 were then used to screen the recombinants in an F_2_ population of 1,054 individuals at the seedling stage. Then, the recombinants were transplanted in the greenhouse for phenotyping and were further genotyped using seven SNP markers (**Figure 3B**). Finally, the *Clwf2* locus was delimited between the markers S_21474486 and S_24306830, with a physical distance of 132.3 kb (Chr06:24,174,486–24,306,830) (**Figure 3B**).

### Several genes in the candidate region are downregulated in white flesh

According to the watermelon reference genome 97103 (v2), the 132.3-kb genome region contains 13 putative genes (**Figure 3C**; **Supplementary Table S4**), 11 of which have been annotated. However, no gene in this region was annotated in the CBP. The 11 annotated genes include two leucine-rich repeat receptor-like kinases (LRR-RLKs) (*Cla97C06G121800* and *Cla97C06G121810*), four chloroplastic proteins (*Cla97C06G121830*, *Cla97C06G121870*, *Cla97C06G121880*, and *Cla97C06G121890*), one cyclin-dependent kinase (CDK)-activating kinase assembly factor (*Cla97C06G121840*), one ATP-dependent DNA helicase (*Cla97C06G121850*), one APETALA2-like ethylene-responsive factor (AP2/ERF) (*Cla97C06G121910*), one heptahelical transmembrane protein (*Cla97C06G121900*), and one histone-lysine *N*-methyltransferase (*Cla97C06G121920*). For the four chloroplastic protein genes, *Cla97C06G121830* encodes a pentatricopeptide repeat-containing protein (PPR), *Cla97C06G121870* encodes a 50S ribosomal protein, *Cla97C06G121880* encodes a DNA-directed RNA polymerase subunit beta, and *Cla97C06G121890* encodes a tetratricopeptide repeat (TPR)-like superfamily protein.

**Figure 4:**
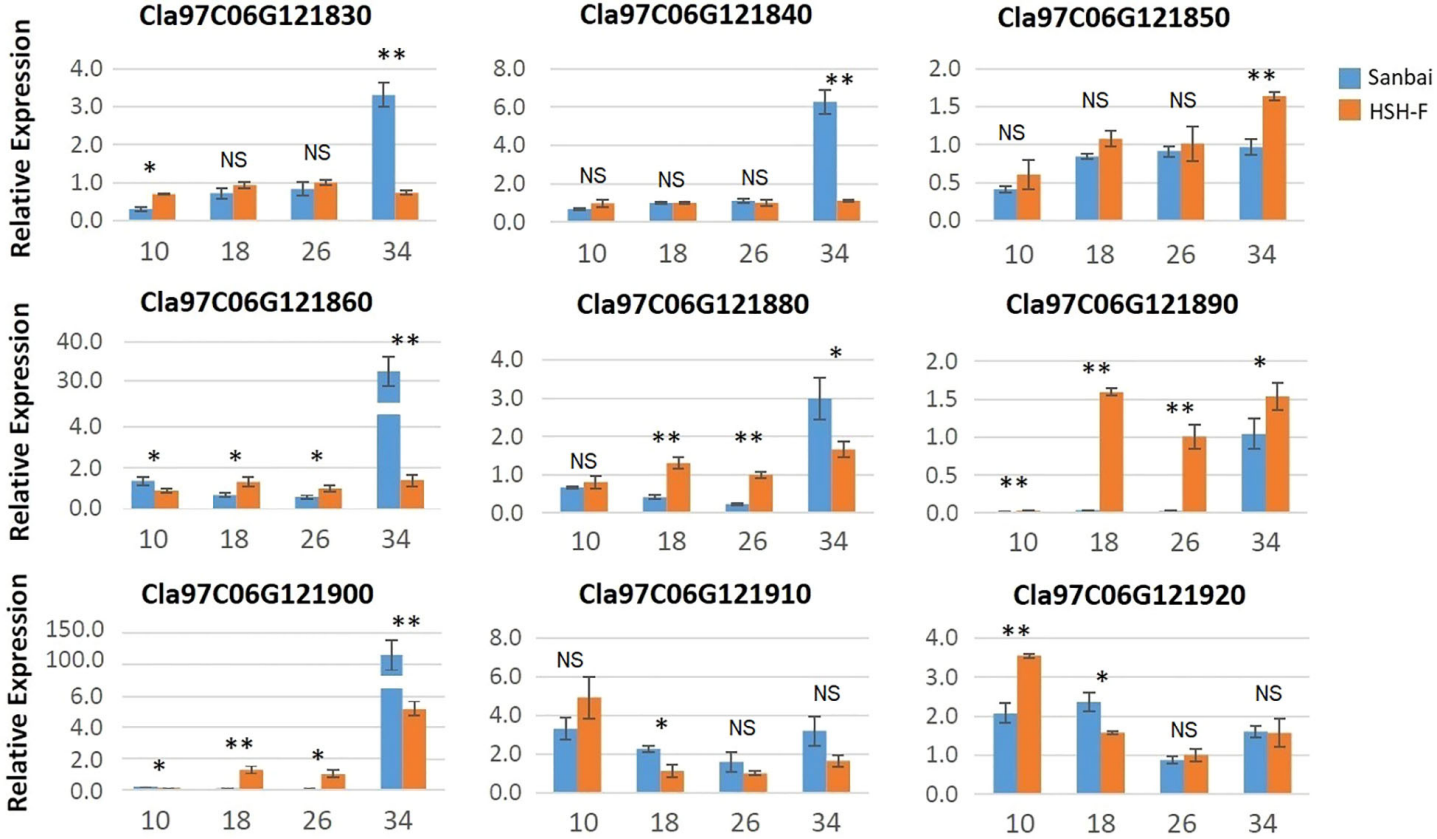
Relative expression of the candidate genes in fruits at different developmental stages of HSH-F and Sanbai. *Numbers on the x*-axis—10, 18, 26, and 34—represent fruits at 10, 18, 26, and 34 days after pollination (DAP), respectively. *Error bars* indicate the mean ± SD (*n* = 3). Significant difference between the two parents at each stage was assessed using Student’s *t*-test. *p < 0.05; **p < 0.01. NS, no significance.

No variant was identified in the candidate region in the comparison of the assembled genome resequencing data of the two parents. The expression levels of the 13 genes in the flesh samples from the two parents at 10, 18, 26, and 34 DAP were then analyzed using qRT-PCR. Among the 13 genes, four (*Cla97C06G121800*, *Cla97C06G121810*, *Cla97C06G121820*, and *Cla97C06G121870*) were only expressed in white flesh at 34 DAP (**Supplementary Figure S2**). Most of the other nine genes showed low or no expression at 10 DAP in both parents, but their expression levels progressively increased during fruit ripening (**Figure 4**). The expression levels of *Cla97C06G121860*, *Cla97C06G121880*, *Cla97C06G121890*, and *Cla97C06G121900* were significantly higher in the canary yellow than in the white flesh at 18 and 26 DAP. Notably, *Cla97C06G121890* and *Cla97C06G121900* showed almost no expression in immature white flesh fruits (10, 18, and 26 DAP). *Cla97C06G121890* is closely related to the *Arabidopsis* (*Arabidopsis thaliana*) REDUCED CHLOROPLAST COVERAGE (REC) proteins, which contribute to establishing the size of the chloroplast compartment and are essential for pigmentation (Larkin et al., 2016). Thus, the white flesh trait in watermelon might be related to the downregulation of the chloroplastic protein.

### Plastid division genes are downregulated in white flesh

**Figure 5:**
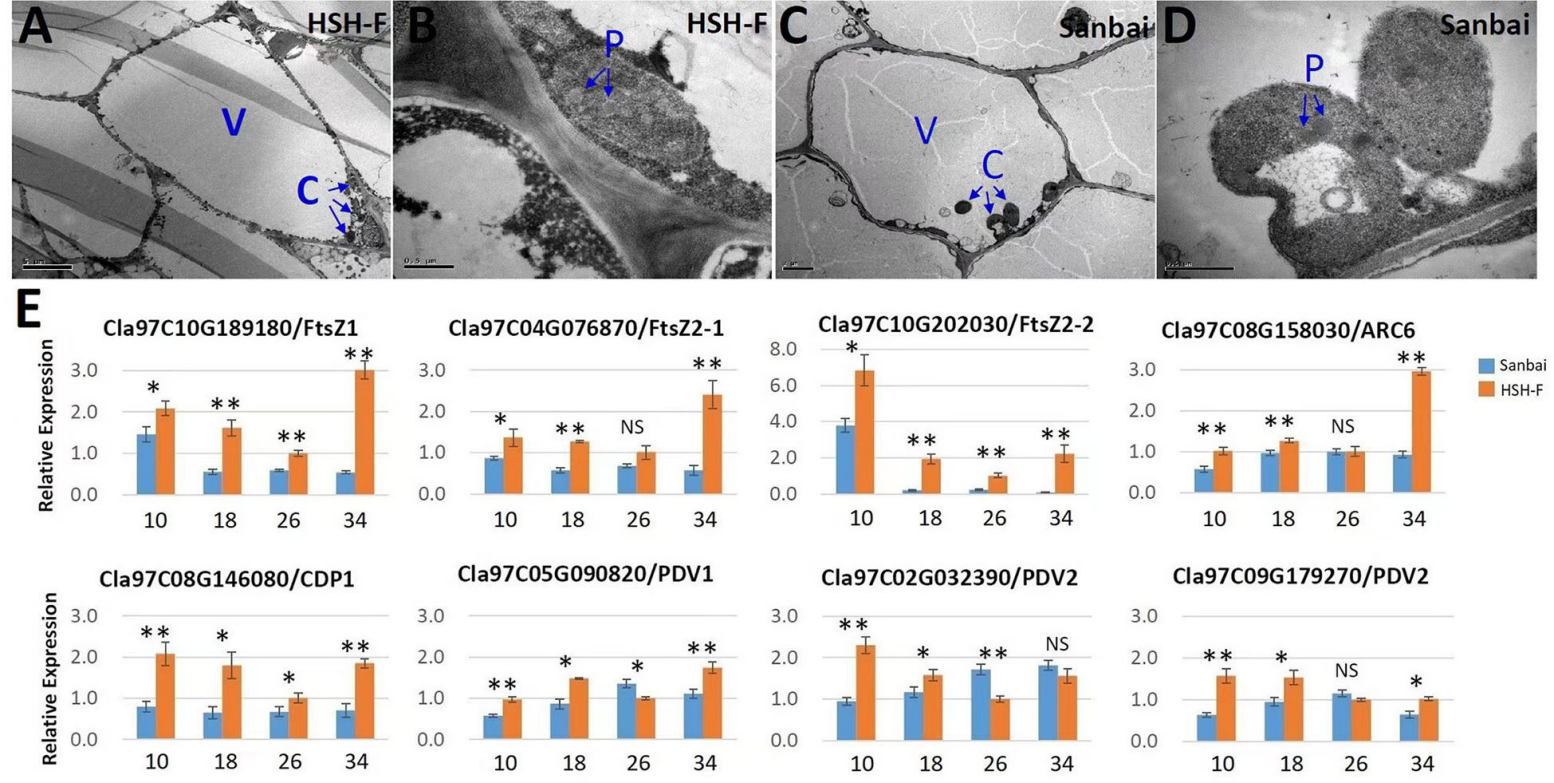
**(A–E)** Transmission electron microscopy of chromoplasts **(A–D)** and relative expression of the chromoplast proliferation-related genes **(E)**. **(A, B)** Micrographs of HSH-F fruit cells at 34 days after pollination (DAP): whole-cell view **(A)** and detailed view of chromoplasts **(B)**. **(C, D)** Micrographs of Sanbai fruit cells at 34 DAP: whole-cell view **(C)** and detailed view of chromoplasts **(D)**. *V*, vacuole; *C*, chromoplast; *P*, plastoglobule. *Error bars* indicate the mean ± SD (*n* = 3). Significant difference between the two parents at each stage was assessed using Student’s *t*-test. *p < 0.05; **p < 0.01. NS, no significance.

Chromoplasts are plastids that accumulate the carotenoid pigments in plants (Egea et al., 2010; Li and Yuan, 2013; Sun et al., 2018). Studies have demonstrated that chloroplasts and chromoplasts are interconvertible (Egea et al., 2010; Li and Yuan, 2013). *RCP2*, a homolog of *Cla97C06G121890* in *Mimulus*, was required for chromoplast development (Stanley et al., 2020). To investigate whether the repressed expression of the chloroplast protein gene affects the development of chromoplasts in watermelon, we examined the ultrastructure of the chromoplasts in flesh cells by TEM at 34 DAP. Notably, rounded and regularly shaped chromoplasts were observed in both canary yellow and white flesh cells (**Figures 5A–D**).

The chromoplast number and size have been reported to affect the accumulation of carotenoids (Sun et al., 2020). We subsequently assessed chromoplast proliferation in white and canary yellow flesh. Because the flesh cell of watermelon is too large to calculate the accurate number of chromoplasts, we then evaluated the relative expression levels of the plastid division genes in fruits of the white and canary yellow flesh parents, including *ClFtsZ1*, *ClFtsZ2*, *ClARC1*, *ClARC6*, *ClCDP*, *ClPDV1*, and *ClPDV2* (Osteryoung and Pyke, 2014; Chen et al., 2019). Most of the chloroplast proliferation genes were significantly downregulated during white flesh fruit development, particularly the *FtsZ* genes (**Figure 5E**). Thus, chromoplast proliferation may be repressed in white flesh watermelon.

### The carotenoid biosynthesis pathway is downregulated in white flesh

**Figure 6:**
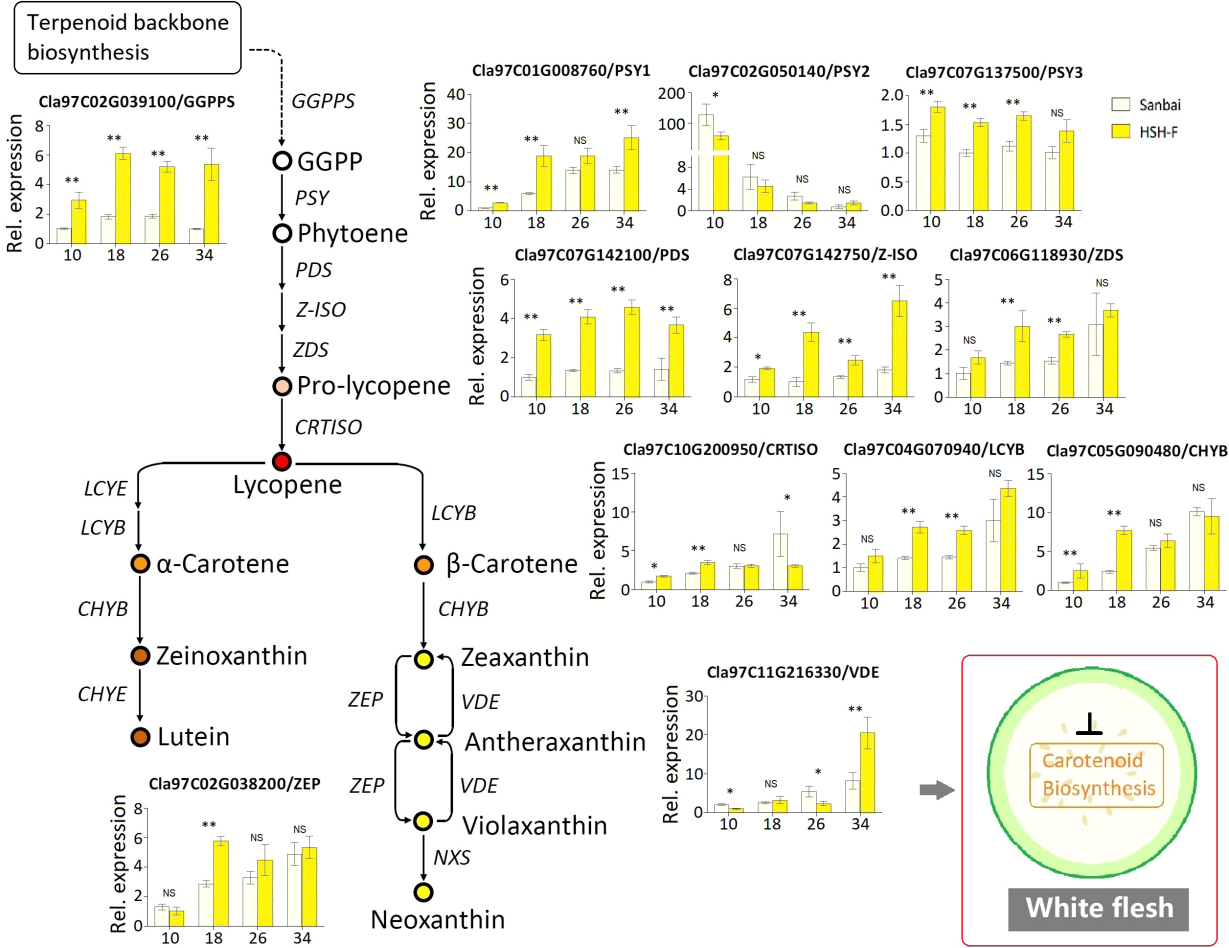
Relative expression of the carotenoid biosynthesis pathway genes in fruits of the canary yellow and white flesh parents. *Numbers on the x*-axis—10, 18, 26, and 34—represent fruits at 10, 18, 26, and 34 days after pollination (DAP), respectively. *PSY*, phytoene synthase; *GGPPS*, geranylgeranyl pyrophosphate synthase; *PDS*, phytoene desaturase; *Z-ISO*, 15-*cis*-ζ-carotene isomerase; *ZDS*, ζ-carotene desaturase; *CRTISO*, lycopene isomerase; *LCYB*, lycopene *b*-cyclase; *CHYB*, carotenoid β-hydroxylase; *ZEP*, zeaxanthin epoxidase; *VDE*, violaxanthin de-epoxidase. *Error bars* indicate the mean ± SD (*n* = 3). Significant difference between the two parents at each stage was assessed using Student’s *t*-test. *p < 0.05; **p < 0.01. NS, no significance.

The C20 GGPP molecule is the direct precursor for carotenoid biosynthesis. We first examined the relative expression level of *GGPPS* (*Cla97C02G039100*) in the fruits of the canary yellow and white flesh parents. The expression level of *GGPPS* in canary yellow flesh was significantly higher than that in white flesh during the whole fruit development stage (**Figure 6**). *PSY* is the primary bottleneck gene in carotenoid biosynthesis (Nisar et al., 2015; Yuan et al., 2015). Three *PSY* genes have been annotated in the watermelon genome: *PSY1* (*Cla97C01G008760*), *PSY2* (*Cla97C02G050140*), and *PSY3* (*Cla97C07G137500*). *PSY1* had a much higher expression level in watermelon fruits compared to *PSY2* and *PSY3* (**Figure 6**). The expression level of *PSY1* gradually increased with fruit ripening, and *PSY1* showed a significantly higher expression level in canary yellow flesh than in white flesh at all developmental stages. Similarly, the *PDS* (*Cla97C07G142100*) and *Z-ISO* (*Cla97C07G142750*) genes showed significantly higher expression levels in canary yellow flesh than in white flesh at all developmental stages. We also examined the expression levels of *ZDS* (*Cla97C06G118930*), *CRTISO* (*Cla97C10G200950*), *LCYB* (*Cla97C04G070940* and *Cla97C10G193770*), *CHYB* (*Cla97C05G090480*), and *ZEP* (*Cla97C02G038200*). These genes exhibited increased expression levels with fruit ripening in both parental lines; however, all genes showed significantly higher expression levels in the canary yellow flesh than in the white flesh at 18 or 26 DAP, the vital stages for pigmentation (**Figure 6**). Thus, the entire CBP is repressed in white flesh.

## Discussion

Carotenoids are important bioactive metabolites that contribute to the beautiful colors of yellow, orange, and red in vegetables, fruits, and flowers and improve the nutritional quality in the human diet. The CBP has been well established, and the core genes and enzymes that catalyze carotenoid biosynthesis and degradation have been cloned and studied in many plants (Nisar et al., 2015; Yuan et al., 2015; Sun et al., 2018). However, regulation of the accumulation of carotenoids, which leads to the varied carotenoid content and composition among or within plant species, is not yet clear. Studies on major horticultural crops, such as tomato, citrus, and melon, have revealed diverse regulation mechanisms of carotenoid accumulation (Kato, 2012; Liu et al., 2015; Feder et al., 2019). Transcriptional regulation of the CBP genes is a major mechanism underlying carotenoid accumulation. Enhanced transcription of the bottleneck gene *PSY* along with *PDS* and *CRSTIO* increased the total carotenoid contents in tomato, watermelon, and red pepper (Grassi et al., 2013; Lv et al., 2015; Berry et al., 2019; Ilahy et al., 2019). A high transcript abundance of *LCYB*/*LCYE* was associated with the high accumulation of carotene in fruits (Lu et al., 2006; Ampomah-Dwamena et al., 2009; Liu et al., 2015). In watermelon, the production of violaxanthin and lutein in yellow flesh was positively correlated with the expression levels of *CHYB* and *ZEP* (Lv et al., 2015; Yuan et al., 2021). In addition, the modulation of carotenogenic enzymes and the regulation of chromoplast biogenesis, development, and differentiation have been demonstrated to strongly affect carotenoid accumulation (Yuan et al., 2015; Sun et al., 2018; Feder et al., 2019). Despite great progress having been made in the research of carotenoid metabolism in plants, little is known about carotenoid regulation in watermelon. In this study, genetic mapping of the flesh color regulation gene was performed using a cross from yellow flesh and white flesh watermelon, and the probable mechanism underlying the white flesh color was elucidated.

Several flesh color loci have been identified in watermelon; however, the inheritance of flesh color, especially white flesh, is not yet clear. *Wf* is the first documented locus for the white flesh trait in watermelon. It is dominant epistatic to yellow flesh (*B*) and red flesh (*b*), as a segregation ratio of 12 white:3 yellow:1 red was obtained in the F_2_ generation of a cross between the white and red flesh inbred lines (Shimotsuma, 1963). Another study using the F_2_ population from a cross between the white (COS) and canary yellow (NC-517) flesh inbred lines suggested that the *C* gene for the canary yellow flesh color is dominant over the white flesh (Gusmini and Wehner, 2006). Recently, genetic inheritance analysis using two segregation populations from crossing Bingtangcui (white flesh) with Xihua (canary yellow flesh) and Sashengnaiyougua (white flesh) with Xinjinlanxuan (canary yellow flesh) has suggested that canary yellow is recessive to white in watermelon flesh (Diao et al., 2021). In this study, the flesh color inheritance analysis using the F_2_ and F_3_ segregation populations produced from the cross between the canary yellow (HSH-F) and white (Sanbai) flesh lines obtained a 3:1 segregation ratio of canary yellow/white flesh (**Table 1**). Consistent with previous studies, the canary yellow flesh contained abundant xanthophyll, whereas white flesh watermelon accumulated trace carotenoids (**Figure 1B** and **Supplementary Table S2**) (Bang et al., 2010; Zhao et al., 2013; Lv et al., 2015; Diao et al., 2021; Yuan et al., 2021). To examine whether the white flesh in Sanbai resulted from the malfunction of the canary yellow flesh locus, we crossed an orange flesh line (CG149), which accumulated massive carotene but lacked xanthophyll (**Figure 1B** and **Supplementary Table S2**), with the canary yellow (HSH-F) and white flesh (Sanbai) lines. Interestingly, both F_1_ hybrids (CG149 × HSH-F and CG149 × Sanbai) had canary yellow flesh, similarly to the F_1_ hybrid that resulted from crossing HSH-F with Sanbai (**Figure 2B**). This result indicated that both Sanbai and HSH-F complement the xanthophyll biosynthesis deficient in the orange flesh line CG149. Thus, the above findings suggest that the *Clwf2* locus for the white flesh color and the *C* locus for the canary yellow flesh are two genetic loci. Moreover, *Clwf2* is different from the *Wf* locus because the white flesh in this study is recessive epistatic to the canary yellow flesh.

The well-assembled genome sequence facilitated the genetic mapping and cloning of the function genes in watermelon. Three flesh color-related loci have been reported on chromosome 6 (Guo et al., 2019; Li et al., 2020; Diao et al., 2021). Li et al. (2020) narrowed a scarlet red flesh color locus (i.e., *Y^scr^*) in a 40-kb region on chromosome 6 that contains five putative genes (*Cla018767*–*Cla018771*). Diao et al. (2021) located a canary yellow flesh locus in a 600-kb region (24.00–24.61 Mb) on chromosome 6. Based on functional annotation and qRT-PCR analysis, the genes *Cla97C06G121680*, *Cla97C06G121700*, *Cla97C06G121890*, *Cla97C06G122090*, and *Cla97C06G121910* were predicted as the candidate genes for the canary yellow flesh in watermelon. In this study, genetic mapping using the canary yellow (HSH-F) and white (Sanbai) flesh lines narrowed the white flesh locus *Clwf2* to a 132.3-kb region on chromosome 6 (Chr06:24,174,486–24,306,830) (**Figure 3**). According to the watermelon reference genome 97103 (v2), the candidate region for the *Clwf2* locus overlapped with the candidate regions for *Y^scr^* and the canary yellow flesh locus. These findings emphasize the important role of this chromosome region in regulating the flesh color in watermelon. Notably, no CBP gene was identified in these candidate regions. The study by Ren et al. (2018) showed that overexpression of the tonoplast sugar transporter gene *ClTST2* (*Cla97C02G036390*) can lead to flesh color development in watermelon. Moreover, the watermelon fruit chromoplast-localized phosphate transporter ClPHT4;2 (*Cla97C10G205070*) was necessary for carotenoid accumulation in fruit flesh (Zhang et al., 2017). These studies concluded that non-CBP genes also play important roles in flesh color regulation in watermelon.

Many transcriptional regulators have been demonstrated to regulate the accumulation of carotenoids in vegetables, flowers, and fruits (Liu et al., 2015; Yuan et al., 2015; Stanley et al., 2020). For example, the MADS-box transcription factor (TF) RIPENING INHIBITOR (*RIN*), STAY-GREEN 1 (*SGR1*), AP2/ethylene response factor-type TF (*RAP2.2*), and phytochrome interacting factor 1 (*PIF1*) regulate the accumulation of carotenoids in tomato fruits by interacting with the *SlPSY1* promoter (Liu et al., 2015). In watermelon, four PPR family genes, which are important RNA-binding proteins in plants, were correlated with flesh color (Subburaj et al., 2020). In the present study, several transcriptional regulators were identified in the candidate region for *Clwf2*, such as the PPR protein (*Cla97C06G121830*), the TPR protein (*Cla97C06G121890*), and AP2/ERF (*Cla97C06G121910*) (**Supplementary Table S5**). Among the TFs, the TPR protein gene (*Cla97C06G121890*) showed almost no expression in immature fruits (10, 18, and 26 DAP) of white flesh watermelon (**Figure 4**). This result is consistent with the study by Diao et al. (2021), in which *Cla97C06G121890* had a much higher expression level in canary flesh than in white flesh watermelon. TPRs are known to mediate protein–protein interactions in diverse biological processes (Zeytuni and Zarivach, 2012). In *Arabidopsis*, the loss of function of the TPR protein gene (*REC*) decreased the chlorophyll content and caused smaller chloroplasts in leaves (Larkin et al., 2016). More recently, the TPR protein gene (*RCP2*) was found to be necessary and sufficient for chromoplast development and carotenoid accumulation in *Mimulus* floral tissues, and the loss-of-function mutation of *RCP2* caused the malformation of chromoplasts and the drastic downregulation of the entire CBP (Stanley et al., 2020). Similar to the study on *Mimulus* flowers, we found that almost all CBP genes were downregulated in white flesh watermelon fruits (**Figure 6**). Thus, the white flesh trait in watermelon is probably related to the repression of the TPR protein gene *Cla97C06G121890*. However, not only *Cla97C06G121890* but also three other genes (*Cla97C06G121860*, *Cla97C06G121880*, and *Cla97C06G121900*) adjacent to *Cla97C06G121890* were downregulated in white flesh fruits compared to canary yellow flesh fruits (**Figure 4**). Thus, further studies investigating the mechanisms underlying transcriptional repression of this genomic region will help in understanding the formation of white flesh in watermelon.

Chromoplasts are the organelles for carotenoid biosynthesis and storage in plant cells, and their number and size are closely related to the accumulation of carotenoids (Li and Yuan, 2013; Sun et al., 2018). In addition to the TPR proteins, the *Or* gene encodes a plastid-associated protein that has been found to increase the accumulation of β-carotene in cauliflower, melon fruits, and tomato flower by triggering chromoplast differentiation (Paolillo et al., 2004; Lu et al., 2006; Chayut et al., 2017; Yazdani et al., 2019). In *Arabidopsis*, *Or* controlled the chromoplast number by specifically interacting with the chloroplast division regulator ACCUMULATION AND REPLICATION OF CHLOROPLASTS 3 (*ARC3*), and overexpression of the plastid division factor *PLASTID DIVISION 1* greatly enhanced the carotenoid accumulation in calli (Sun et al., 2020). Tomato mutants with enhanced plastid number and size (i.e., *hp1*, *hp2*, and *hp3*) had higher levels of carotenoids in fruits (Liu et al., 2004; Kolotilin et al., 2007; Galpaz et al., 2008). In the present study, rounded and regularly shaped chromoplasts were observed in both canary yellow and white flesh fruit cells (**Figures 5A–D**), indicating that the lack of carotenoid in white flesh watermelon did not result from the malformation of chromoplasts. However, the qRT-PCR analysis showed that several key genes associated with plastid division, such as the *FtsZ* genes (*Cla97C10G189180*, *Cla97C04G076870*, and *Cla97C10G202030*), were significantly downregulated in white flesh watermelon fruits (**Figure 5E**). Thus, whether chromoplast differentiation is inhibited in white flesh watermelon and subsequently blocks carotenoid accumulation is worth further study.

In conclusion, we characterized a *Clwf2* locus that determines the white flesh trait in watermelon. From the results of the carotenoid content determination, genetic mapping, expression level evaluation, and TEM analyses, we conclude that *Clwf2* regulates the white flesh trait probably by repressing the expression of the TPR protein gene (*Cla97C06G121890*), which further inhibits the proliferation of chromoplasts and causes downregulation of the CBP genes, blocking the accumulation of carotenoids.

## Data availability statement

The data presented in the study are deposited in the NCBI repository, accession numbers SAMN22357443 (HSH-F, https://www.ncbi.nlm.nih.gov/biosample/?term=SAMN22357443) and SAMN22357441 (Sanbai, https://www.ncbi.nlm.nih.gov/biosample/?term=SAMN22357441).

## Author contributions

ZD and LY designed the study. LY, WZ, ZC, and NW contributed to the experiments. LY and YZ analyzed the experimental data. ZD provided the seeds for the experiment. LY wrote the manuscript. YZ and YW revised the manuscript. All authors contributed to the article and approved the submitted version.
