# Supplementary figures and images for: Genetic mapping of a single nuclear locus determines the white flesh color in watermelon (Citrullus lanatus L.)
Source: Front Plant Sci. 2023 Feb 7;14:1090009. doi: 10.3389/fpls.2023.1090009 (PMC9941332; doi:10.3389/fpls.2023.1090009)

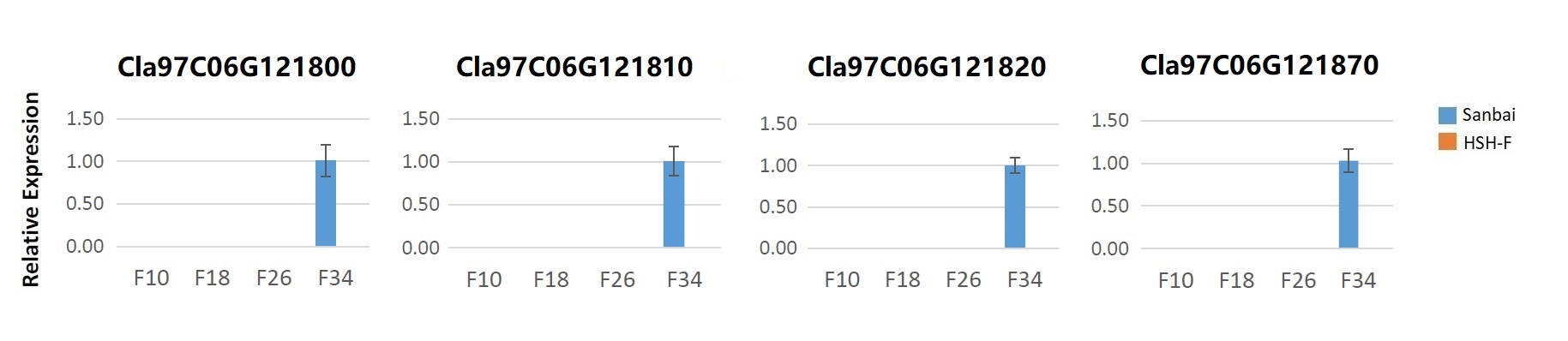

Supplement: Supplementary file 1 [file Image_1.jpeg]
